# Engineering Strategies for Fungal Cell Disruption in Biotechnological Applications

**DOI:** 10.1002/elsc.70061

**Published:** 2025-12-01

**Authors:** Bhagyeshri Ulhas Mantri, Maliheh Vahidinasab, Sonja Berensmeier

**Affiliations:** ^1^ Chair of Bioseparation Engineering TUM School of Engineering and Design Technical University of Munich Garching Germany; ^2^ Munich Institute for Integrated Materials Energy and Process Engineering Technical University of Munich Garching Germany

**Keywords:** bead milling, chemical, cell disruption, electrical, enzymatic, fungi, fungi cell structure, high pressure homogenization, physical, ultrasound

## Abstract

Fungal cell disruption plays a critical role in unlocking a wide range of high‐value intracellular products such as lipids, proteins, pigments, and bioactive compounds. However, lysing fungal cells is far more challenging than breaking bacterial or algal cells due to their robust and highly structured cell walls. These biological barriers demand a tailored and strategic approach depending on the fungal species, cell morphology, and downstream processing requirements. This review explores the various mechanical and non‐mechanical methods used to disrupt fungal cells, beyond outlining the core principles behind each method, the engineering and process factors that influence their performance are emphasized. A comparative analysis is provided, focusing on key parameters like disruption efficiency, scalability, cost‐effectiveness, and environmental impact. The review also sheds light on emerging hybrid and integrated approaches, the role of pre‐treatment or co‐treatment strategies, and the potential for greener and more sustainable alternatives aligned with circular bioeconomy goals. Ultimately, this review aims to serve as a guide for researchers, bioprocess engineers, and industry professionals seeking to optimize fungal bioproduct extraction in a way that is not only technically sound but also economically viable and environmentally responsible, paving the way for more efficient, scalable, and sustainable fungal‐based biomanufacturing.

## 1 Introduction

Fungi have played a crucial role in human life for centuries, from traditional uses in food fermentation and beverages to modern industrial biotechnology. Their importance in biotechnology continues to grow, with an expanding range of applications across food production, pharmaceuticals, agriculture, materials science, and environmental sustainability. Well‐known examples include *Aspergillus niger* for production of more than 90% of global citric acid production and *Penicillium chrysogenum* for penicillin synthesis, showing the diversity and industrial relevance of fungal species [1, 2, 3, 4, 5].

Taxonomically, fungi represent one of the most diverse kingdoms of life. While traditional classification divided them into five major phyla, advances in molecular phylogenetics have expanded the classification to nine distinct fungal phyla, including Ascomycota, Basidiomycota, Mucoromycota, and Chytridiomycota, among others [6]. Fungi are estimated to comprise around 1.5 million species, although only approximately 80,000 have been described [2, 7]. Of these, only a small number have been harnessed for industrial biotechnology, with *Saccharomyces cerevisiae, Yarrowia lipolytica*, and *Aspergillus* spp. among the most widely used. Therefore, there is a significant opportunity to harness other strains for future biotechnological applications for the production of valuable metabolites with potential applications in different fields.

**FIGURE 1:**
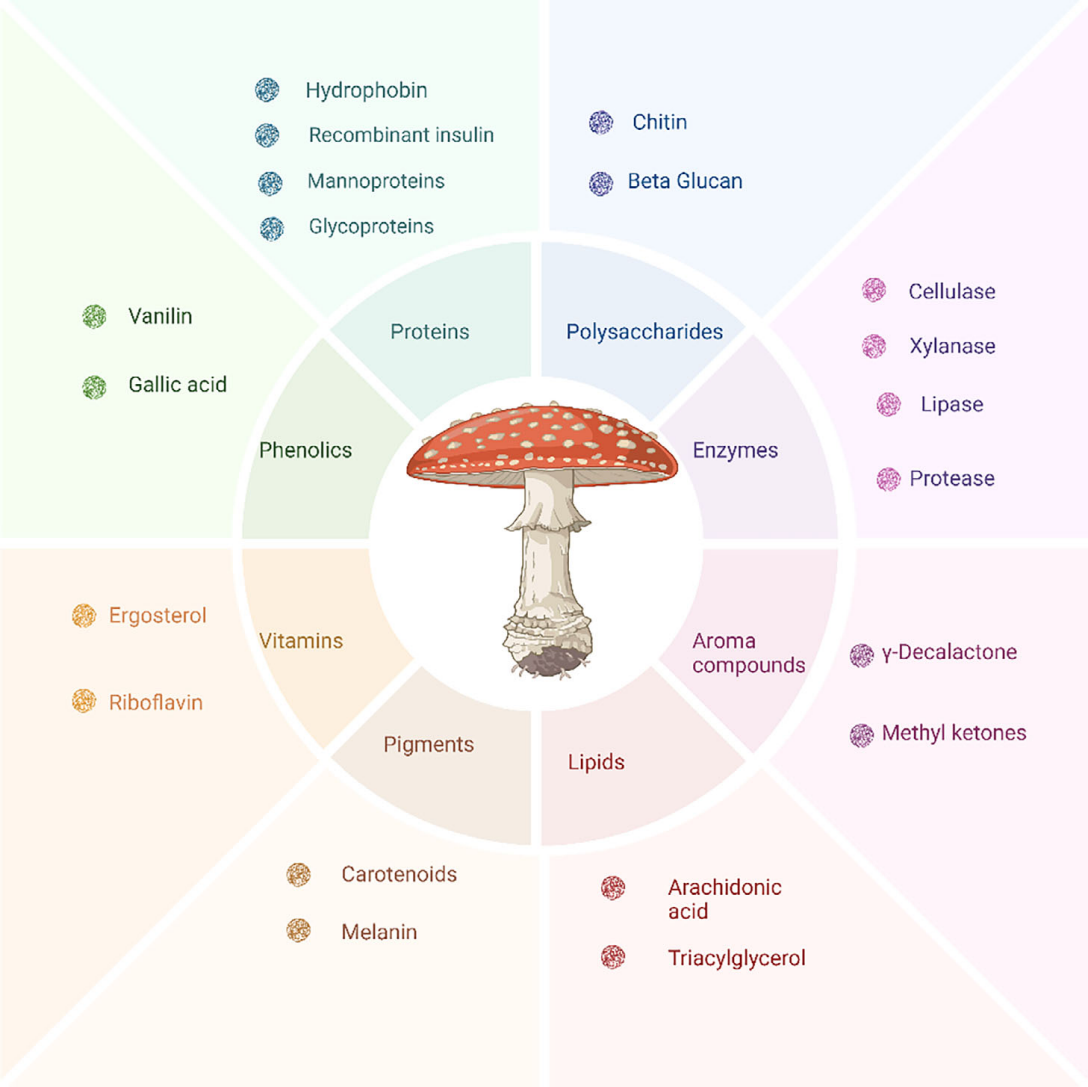
Overview of intracellular products from fungi.

Fungi exhibit diverse morphologies, ranging from unicellular yeasts to multicellular filamentous forms with complex hyphal networks. This morphological flexibility contributes to their ecological success and biotechnological utility. Yeasts such as *S. cerevisiae* are widely used for ethanol and enzyme production, while filamentous fungi like *Aspergillus*, *Trichoderma*, and *Ganoderma* spp. are increasingly exploited for enzymes, organic acids, secondary metabolites, and alternative protein products. Oleaginous fungi such as *Mortierella* spp. and *Y. lipolytica* are particularly valued for intracellular lipid production [1, 2, 3, 4, 5]. The fungal cell factory concept highlights their capacity to produce a wide range of intracellular metabolites, including enzymes, antibiotics, pigments, bioactive peptides, and nutraceutical compounds, as summarized in Figure 1 [8, 9, 10]. Many fungal‐derived extracellular secondary metabolites, such as polyketides, terpenoids, non‐ribosomal peptides, and alkaloids, exhibit pharmacologically relevant activities, including antimicrobial, antioxidant, and anticancer effects. Fungal pigments are also gaining attention as natural colorants for food and feed [11]. However, accessing these valuable intracellular products requires efficient cell disruption methods, which are critical for product recovery due to the rigid and highly variable nature of fungal cell wall structures.

The fungal cell wall is composed mainly of chitin, β‐glucans, and glycoproteins, which confer mechanical strength and resistance to disruption. Additionally, morphological features such as hyphal aggregation, pellet formation, and the development of fruiting bodies further complicate downstream processing. For example, in submerged fermentation, filamentous fungi tend to form pellets through coagulative or non‐coagulative mechanisms, affecting mass transfer and extraction efficiency [12, 13]. Due to the complexity and diversity of fungal cell structures, particularly in filamentous and oleaginous species, the choice of cell disruption strategy significantly influences product yield and recovery. This becomes especially relevant in the context of industrial‐scale bioprocesses aiming to valorize fungal biomass into biofuels, biochemicals, and nutritional metabolites.

This review provides a comprehensive overview of the current and emerging methods used for fungal cell disruption, focusing on mechanical, physical, chemical, and enzymatic techniques. Additionally, the relevance of each method, depending on the fungal morphology, the target product, and the scale of application, is discussed. The goal is to facilitate method selection for researchers in the downstream processing of the fungal fermentations. The next sections of this review provide an in‐depth comparison of fungal cell wall composition of different fungi, followed by a detailed evaluation of cell disruption methods, advantages, and limitations.

## 2 Structural Complexity of Fungal Cells

Scientific interest in fungal cell walls started back in 18th century. However, the full understanding of fungal cell wall biosynthesis, structure, and function remains elusive [14, 15]. The major challenge lies in the vast diversity of fungi. There are more than a thousand different fungal species, but only a few have been studied.

**FIGURE 2:**
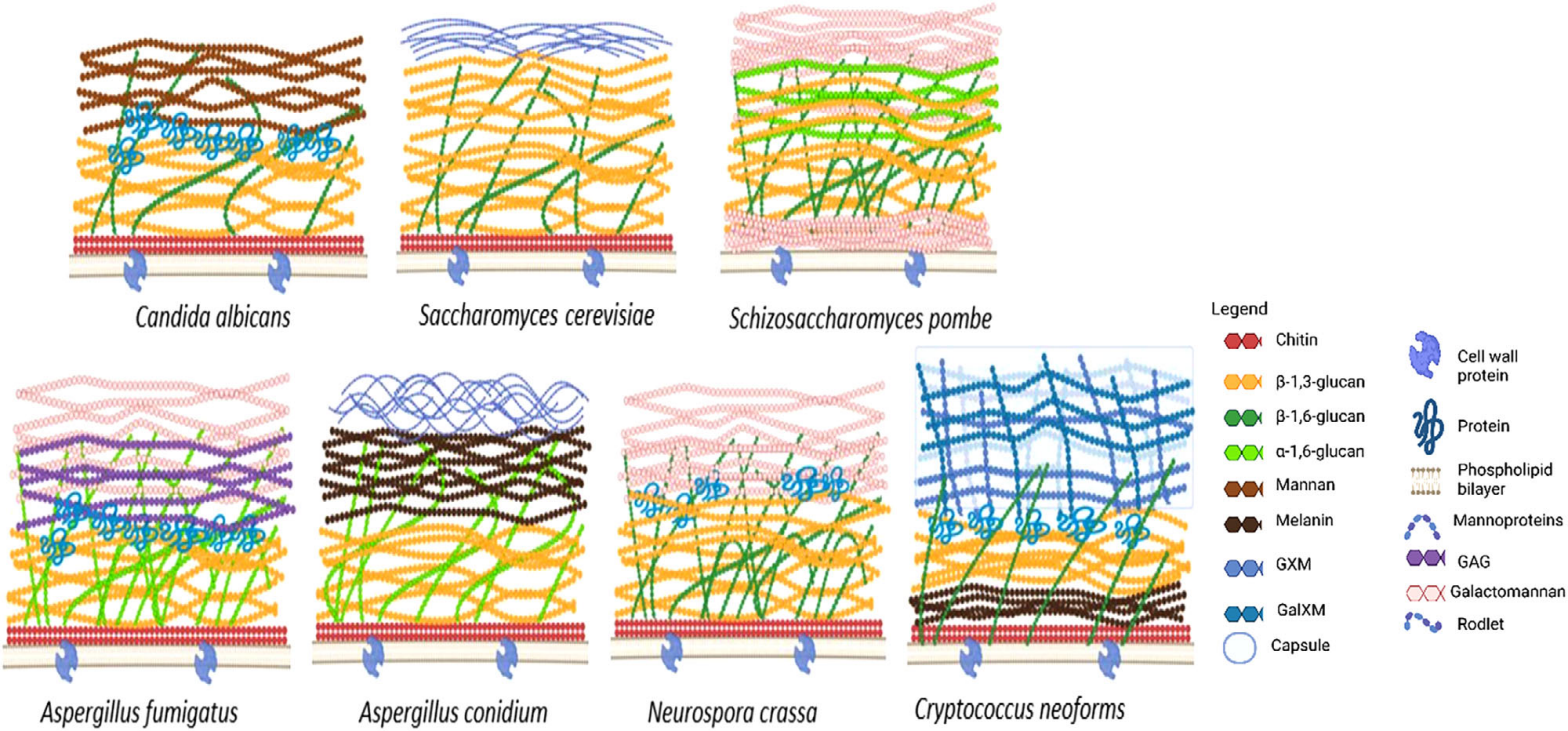
Cell wall structure of different fungi [15, 18, 19, 20].

In general, the fungal cell wall can be divided into two domains: (a) structural components such as chitin and β‐1,3 glucan, which are commonly present in all species, and β‐1,4 glucan and β‐1,6 glucan, which vary between species; and (b) matrix or intrastructural components including mannoproteins, galacto‐mannoproteins, xylo‐mannoproteins, glucurono‐mannoproteins, and α(1–3) glucan, which exhibit species‐specific variability [14, 16]. These components are often cross‐linked through extracellular interactions, forming a robust and highly structured matrix. The outer layers of the cell wall, in particular, vary considerably among species [17]. Figure 2 shows the different layers and components of different fungi.

The complex cell wall structure provides fungi with mechanical strength, elasticity, and morphogenetic stability, while also contributing to hydrophobicity and adhesion, which are important for processes such as biofilm formation [15, 16]. As fungi are chemoheterotrophs, the cell wall also acts as a selective permeability and porosity barrier that helps it to take up nutrients from the environment [21]. The glycoproteins present in the fungal cell walls are N‐ or O‐glycosylated and may include glycosylphosphatidylinositol (GPI) anchors, which contribute to cell wall integrity. β‐glucan represents a major component comprising 30% to 90% of the cell wall, depending on the species, and typically consists of β‐1,3; β‐1,4 glucan; or β‐1,6 glucose in its chains [14, 16, 22]. Chitin, a polymer of β‐1,4‐linked N‐acetylglucosamine residues, is found in all species in smaller amounts, around 2% in yeast and up to 15% in filamentous fungi [22, 23].

**FIGURE 3:**
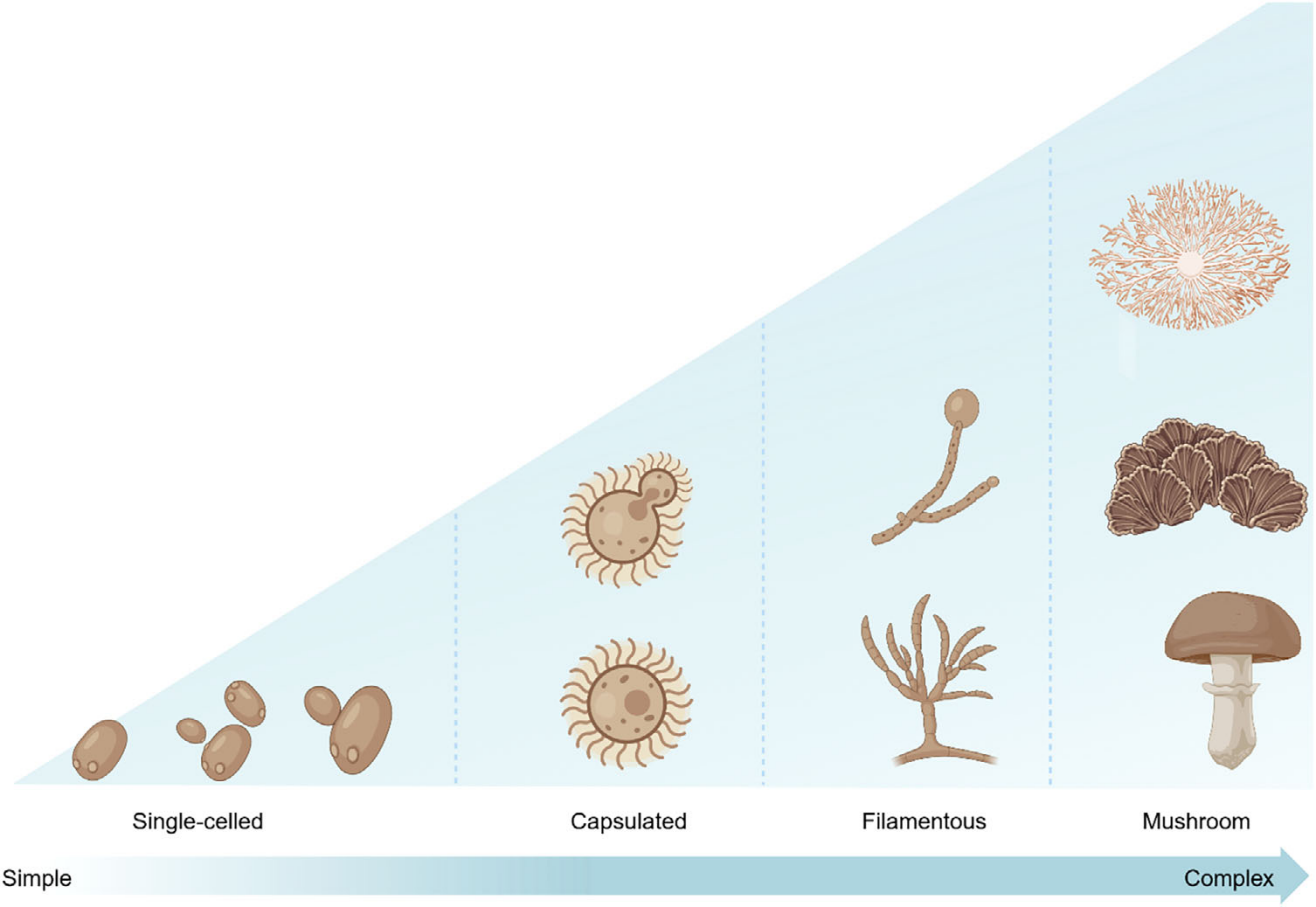
Cell morphology of different fungi.

So far, an in‐depth study of cell wall structure and components has been conducted for only a few fungi, including *S. cerevisiae*, *Candida albicans*, *Aspergillus fumigatus*, *Neurospora crassa*, *Schizosaccharomyces pombe*, and *Cryptococcus neoformans*. Out of which *C. neoformans* belongs to basidiomycete division, whereas others are ascomycetes [23]. Most of the studies on cell walls are from the vegetative state of the cells, and it would have been great to have them from varied stages of life, as cell wall composition keeps changing with the aging of the cells [16]. Cellulose is also present in some of the oomycetes, whereas in some amount, amino acids, lipids, N‐acetylglucosamine, uronic acid, hydrophobins, and melanins are also present in most fungi [14]. Figure 3 illustrates the increasing morphological complexity of fungi, progressing from simple unicellular yeast forms, through spore‐producing structures and branching mycelia, to complex multicellular fruiting bodies such as mushrooms.

As fungi are biotechnologically important to humans, it is necessary to know if there is any relation between species‐specific cell wall structure and its disruption. It is also important to know more about the fungus‐specific polysaccharides as well as the protein context by disrupting the cell wall. This knowledge will help to improve the extraction of various compounds such as protein, chitin, beta‐glucan, lipids, ergosterol, carotenoids, and so on. Most of the disruption methods are studied for well‐known fungi like *S. cerevisiae* or *A. niger*, but not on rare or uncommon fungi.

## 3 Cell Disruption Technique

**FIGURE 4:**
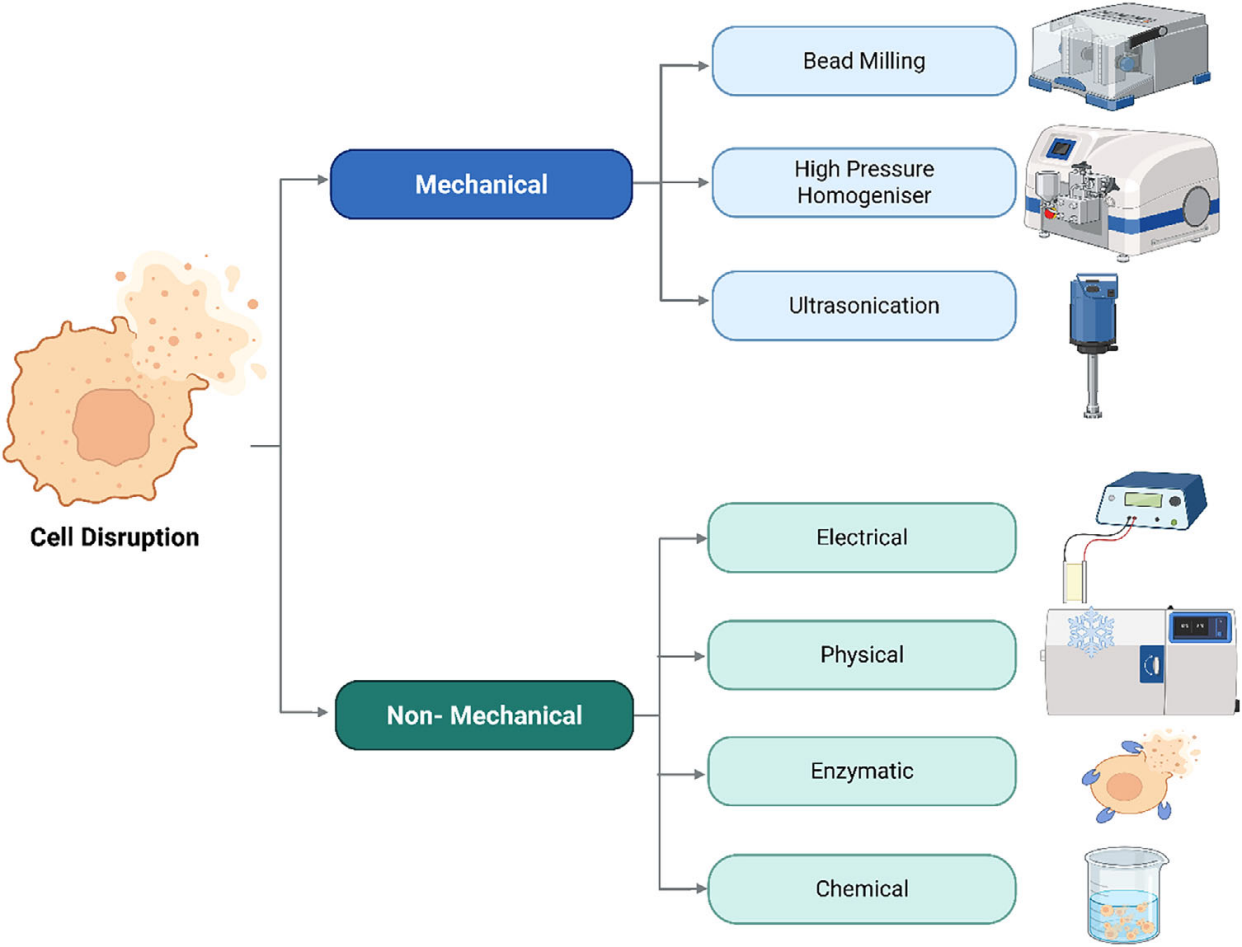
Overview of fungi cell disruption methods.

Cell disruption is a critical step in the recovery of the intracellular products from microorganisms, including fungi. The choice of disruption method depends on several factors such as structure and morphology of the fungal species, location of the target product within the cell, and sensitivity of the product to mechanical or chemical stress. For fungi, which often possess rigid and complex cell wall structures, especially in filamentous and oleaginous forms, the disruption strategy must be carefully selected to ensure efficient release while minimizing product degradation and not too many cell debris to avoid further subsequent difficulty in solid removal process. Cell disruption techniques can be divided into two main categories: mechanical and non‐mechanical methods, as it is shown in Figure 4.

Mechanical methods physically break the cell wall using forces such as shear, pressure, or vibration. Common mechanical techniques include high‐pressure homogenization, ultrasonication, and bead milling. These methods are generally effective for a wide range of fungal species, particularly those with tough cell walls, and are scalable for industrial applications.

Non‐mechanical methods include electrical, physical, enzymatic, and chemical approaches [18, 24, 25]. Some of these techniques are directly combined with extraction techniques for increasing the solubility of defined target molecules and combining their further separation from undesired molecules. These methods are often more selective or milder than mechanical ones, making them suitable for sensitive products or for applications where purity is essential. However, they may be more costly or slower, and their effectiveness can vary significantly depending on fungal species and growth conditions.

### 3.1 Mechanical Cell Disruption Methods

Mechanical disruption methods rely on physical mechanisms such as shearing forces, which are employed to disrupt the cell wall of the organism by the use of some mechanical devices. They are nonspecific and severe disruption methods, making them particularly suitable for organisms with tough, rigid cell walls, such as many fungi. Their effectiveness, scalability, and adaptability to different bioprocessing setups make them widely used in fungal biotechnology [26].

#### 3.1.1 Bead Milling

Bead mills or ball mills are one of the most established mechanical methods used for microbial cell disruption. Originally developed for grinding hard materials, but later used in biotechnology for disrupting the cell walls of different microorganisms, including gram‐positive bacteria, algae, and fungi, particularly yeast cells. More recently, it has been applied to filamentous and oleaginous fungi as well [27, 28].

A typical bead mill usually consists of a grinding chamber, which may have temperature control if required, and an agitating disk, which is centrally or eccentrically mounted to the chamber through the motor‐operated shaft. During the disruption, the chamber is filled with the grinding media, that is, beads made of steel, zirconium, ceramic, or glass. Agitation causes the beads to collide with the fungal cells, applying shear and compression forces that disrupt the cell walls.

**FIGURE 5:**
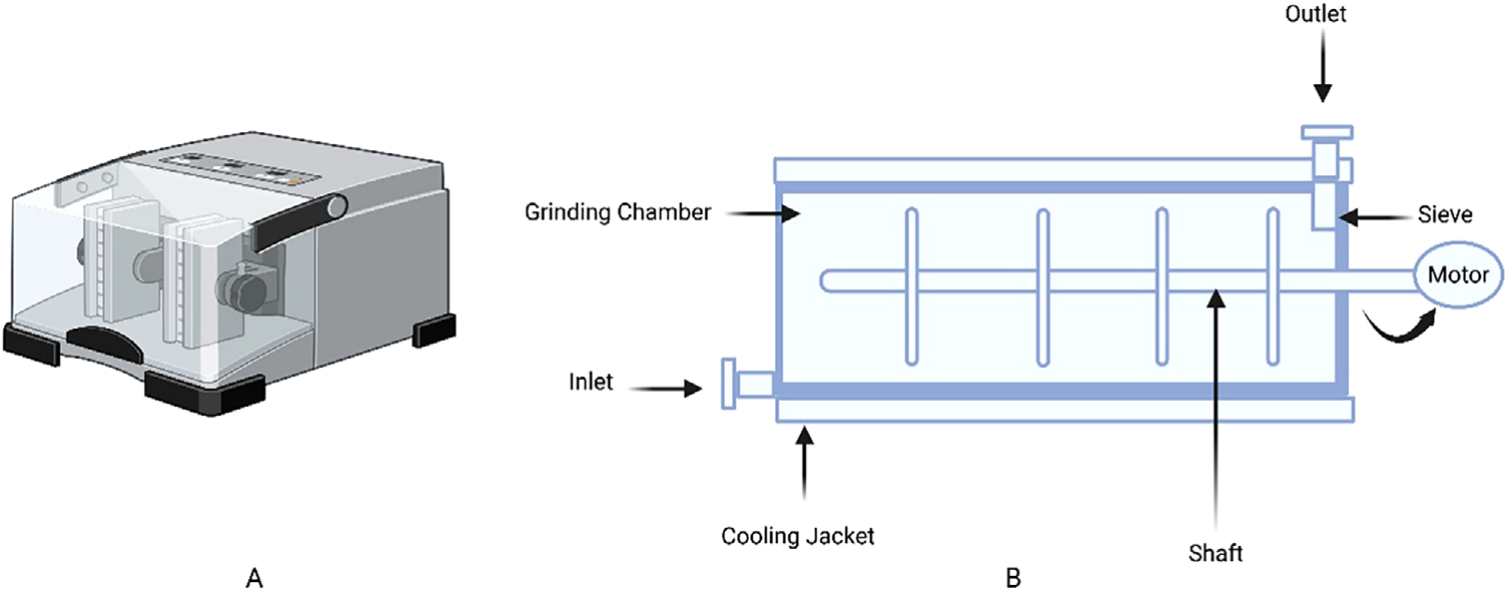
Bead mill equipment. (A) Laboratory scale and (B) Industrial scale.

**Disruption parameters and mechanism**: The efficiency of bead milling and the yield of desired molecule released per gram of used fungal biomass in bead milling can be affected by different parameters such as bead size and density, bead‐to‐cell ratio, agitation speed, temperature, and processing time. The bead milling equipment used in laboratory and industry is shown in Figure 5.

Smaller beads generate higher collision frequency, while larger beads deliver greater impact force. The balance between these determines the optimal disruption conditions for different fungal types.

Disruption in bead mill is often modeled using first‐order kinetics [29]. In batch bioprocesses, the release of intracellular protein (*R*) over time (*t*) is represented by:

$$\ln\left(\frac{R_{\max}}{R_{\max}-R}\right)=kt$$

where the *R*_max_ is the maximum amount of protein that can be released, *R* is the protein released at time *t*, *k* is the disruption rate constant, and *t* is the duration of the cell's treatment.

For continuous operation mode, assuming back mixing and multiple stirrer stages, the equation can be stated as follows:

$$\frac{R_{\max}}{R_{\max}-R}={\left(1+\frac{kT}{j}\right)}^{j}$$

Herein, *T* represents the mean residence time in the mill (*T = V/Q*, with *V* as the total volume of the mill *Q* as the total throughput), and *j* is the number of the bead mills in the series [29].

Later studies represented the kinetics in terms of number of cells disrupted, which can be stated as:

$$\ln\left(\frac{C_{0}}{C_{0}-C}\right)=kt$$

whereas *C_0_* is the initial number of cells, *C* is the number of cells disrupted at the given time *t*, and *k* is the disruption rate constant. The disruption efficiency depends on various operational parameters such as agitator speed, bead loading, and bead diameter or size. The disruption rate constant *k* is influenced by the agitator's peripheral velocity *µ*. However, its dependency on *µ* decreases as *µ* increases [30, 31].

k=Kμ

The disruption efficiency increases with agitator speed. In Annu prototype mill featuring a bead inlet and a cooling jacket, increased agitation speeds from 1000 to 4000 rpm resulted in higher protein and enzyme release [32]. To better study the cell disruption dynamics, the concept of effective disruption volume, also known as active volume, was introduced [30, 31]. This volume represents the space between colliding beads where actual cell disruption occurs [33].

**FIGURE 6:**
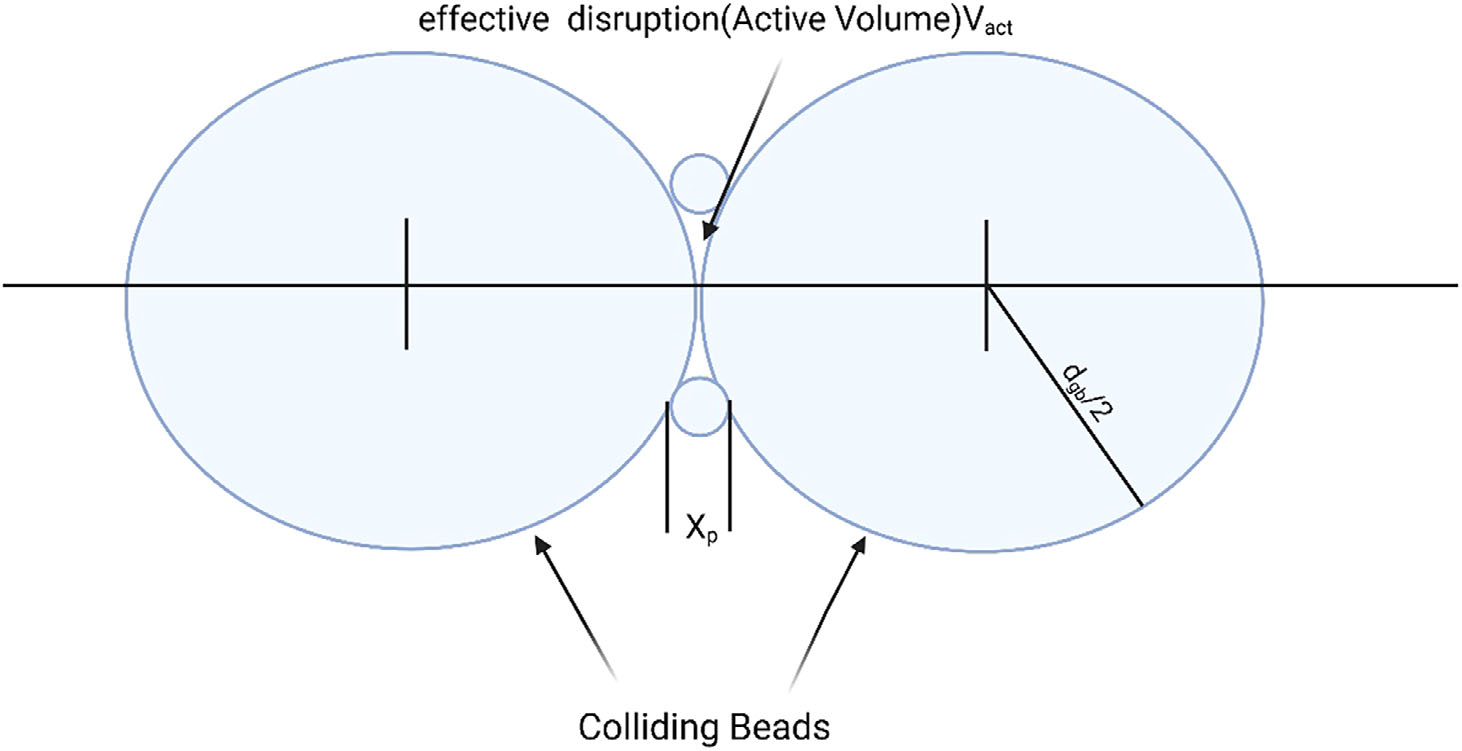
Colliding beads in bead mill [30, 31].

This led to a new equation:

k=fβ

wherein the f is the collision frequency of the beads per unit volume and β is the effective disruption volume, which increases with bead size (Figure 6).

Since the collision frequency *f* is also dependent on the peripheral velocity *µ*, the disruption rate *k* is inherently tied to both bead size and bead loading. This relationship introduces the concept of critical velocity, a threshold velocity at which beads collide frequently but without achieving cell disruption. For cell disruption to happen, the peripheral velocity has to be greater than the minimum velocity. Additionally, the rate of protein release is influenced by bead loading (i.e., the ratio of bead volume to the total the grinding chamber volume). The rate constant can also be expressed as:

$$k=c\;\alpha^{2}$$

where *α* is bead loading and *c* is a constant dependent on the peripheral velocity *µ* [30, 31].

The effect of cell concentration on disruption efficiency remains contradictory. Some studies reported that it affects cell disruption, while others suggest minimal or no impact. For instance, yeast disruption appears largely unaffected with low concentrations ranging from 0.25% to 20% on a dry weight basis. However, at high concentration of 35% to 40% wet basis, an increase in cell disruption and protein release has been observed. In contrast, for some filamentous fungi, cell concentration in the range of 12.5% to 87% wet biomass shows either no effect or even a negative effect on disruption efficiency [27, 29, 31, 34].

The disruption rate constant *k* often increases with the cell concentration, as shown in yeast suspension from 0.002 to 0.17 g dry weight/mL, although this affects process dynamics more than the disruption mechanism itself [35]. Similarly, larger cell sizes have been associated with increased disruption rates, following the first‐order kinetics [36].

Bead diameters and loading are also critical variables. Beads ranging from 0.1 to 1.5 mm are typically used. It is seen that the disruption process is faster when the beads are smaller, but the small beads tend to float and do not retain properly in the grinding chamber, whereas the larger beads are more stable but may be less effective in transferring energy and may not disrupt the cells efficiently. The number of beads also varies with size—higher for smaller beads, lower for larger ones. For fungi, glass or zirconium beads between 0.5 and 1.5 mm have proven effective, with biomass‐to‐bead ratios between 1:1 and 1:2 (w/w) yielding favorable results. However, optimal conditions vary by fungal species due to differences in cell wall structure [37, 38].

Heat generated during bead milling can degrade heat‐sensitive products. To avoid the degradation, removing the heat by adding a cooling liquid, like saline water or brine, and circulating the liquid through a jacket around the mill are used to maintain temperature control.

The disruption rate or effect also depends on the number of passes, which can be related to the continuous processing. For instance, complete disruption of *Neurospora sitaphila* occurred after two passes, whereas for *Pichia pastoris*, it required three passes [34, 39].

Disruption kinetics can be further described using stressing energy (SE) and frequency (SF) [33]. *SE* is defined as the highest possible kinetic energy transferred to particle during bead contact:

$$\mathrm{SE}=\frac{1}{2}\;m_{gb}{v^{2}}_{gb,rel}$$

where *m_gb_* is the mass of grinding beads and *V_gb_* is the relative velocity of grinding beads.

*SF* is proportional to collision frequency and the active disruption volume *V_act_* can be expressed as the following equation.

$$\text{SF}\propto \omega_{cyc}\varphi \frac{1-\varepsilon }{d_{gb}}$$

where *ω_cyc_* is angular velocity of the stirrer, *φ* is the filling degree of the grinding media, ϵ is the porosity of the bulk of grinding media at rest, and *d_gb_* is bead diameter (Figure 6).

Both protein and enzyme release kinetics during bead milling follow first‐order kinetics. Enzyme release can be stated as

$$A_{D}=A_{M}\left(\frac{C}{C_{0}}\right)$$

where *A_D_* is the enzyme released, *A_M_* the maximum releasable enzyme, *C* the number of disrupted cells at time *t*, and *C_0_* is the initial number of cells [40]. Heim [35] studied the effect of cell breakage in terms of linear differential equation, which suggested that generating a linear differential equation for modelling the process is not favorable, whereas later it was proven that it can be done, and the cell disruption in whole as well as in fractions follows the first‐order linear differential equation mentioned in the above equation. The rate constant for protein and cell disruption differs slightly, and earlier is slightly lower than the latter. As well, this model is beneficial for the cells whose size is spherical rather than elliptical.

In summary, bead or ball milling has been extensively applied to yeast, where disruption kinetics are well‐characterized and typically first‐order. However, filamentous fungi and other types with complex morphology remain underexplored. Their rigid cell walls and diverse shapes complicate the generalization of the condition required for complete disruption or product release and require species‐specific optimization. Some comparative analyses of fungal cell disruption using bead milling are presented in Table 1.

**TABLE 1:** Comparison of bead milling strategies for cell disruption in yeasts and filamentous fungi.

Cell type	Fungal species	Target product	Bead material	Bead size (mm)	Bead loading (% or biomass: bead ratio)	Agitation (m/s)	Temperature control (Yes/No)	Disruption efficiency/Yield (%)	Time (min)	Ref.
Filamentous	*Pycnoporus cinnabarinus*	Protein, glucose‐6‐phosphate	Glass	0.40–0.5 mm	60 %	NM^a^	Yes	100%	10 min	[27]
Unicellular	*Rhodotorula glutinis*	Carotenoids	Glass	1 mm	1:1	NM	Yes	89.14%	20 min	[28]
Unicellular	*Saccharomyces cerevisiae*	Beta glucan	Zirconium glass	0.5–1 mm	57 %	NM	No	61% to 64%	30 min	[41]
Unicellular, oleaginous yeast	*Rhodotorula* sp.	Lipids	Glass	0–0.6 mm	1:10	6.5 m/s	No	23.5%	6 min	[42]
Unicellular, oleaginous yeast	*Lipomyces kononenkoae*	Lipids	Glass	0.5–0.6 mm	1:16	6.5 m/s	No	35.8%	6 min	[42]
Unicellular	*Candida guilliermondii*	Glucose‐6‐phosphate	Glass	0.5 mm	1:1	NM	No	NM	5 min	[37]

^a^Not mentioned.

#### 3.1.2 High‐Pressure Homogenizer

In a high‐pressure homogenizer (HPH), the cell suspension is passed through the nozzle or valve at a high pressure and then to the next chamber at a low pressure, which causes the cell suspension to disrupt under the shear forces, turbulence, pressure drop, and cavitation. It has been used for emulsification in industry but also for the disruption of cells. For the filamentous fungi, the use started not long enough back. The pressure they operate at is 1500 bar; for ultra‐high, it can be up to 4000 bar.

Disruption efficiency depends on several variables, such as pressure, time, temperature, flow rate, number of valves/passes, valve design, and impingement geometry.

The cell disruption efficiency in a homogenizer can be modeled using the equation proposed by Hetherington [43] as follows:

$$\ln\left(\frac{R_{\max}}{R_{\max}-R}\right)=kNP^{a}$$

where *R*_max_ is the maximum amount of protein release, *R* is the protein release at a given point of the process, N is the number of passes, and *P* is the pressure. The constant a is the measure of cell resistance to disruption and is organism‐specific and k is the specific rate constant.

The value of a is found to be 2.9 for Saccharomyces cerevisiae below 50 MPa and 1.7 for *Candida utilis* in continuous cultivation at 50–125 MPa. Above 50 MPa, a deviates from first‐order behavior, decreasing with pressure [43, 44].

For filamentous fungi like *Rhizopus nigricans*, *R*_max_ was achieved within three passes at a pressure above 10 MPa. Similar outcomes were reported for pelleted cultures at 5 to 20 MPa, indicating pressure dependency for both filamentous fungi and unicellular yeast [45].

The effect of cell concentration on the rate constant was studied for *R. nigricans* in the range of 8 to 22 g/L dry weight and for pelleted cells up to 30 g/L. No significant impact on disruption efficiency was observed below clogging limits. In S. cerevisiae, concentrations up to 600 g/L (packed yeast) were studied with comparable outcomes [43, 45, 46, 47].

The rate constant k is influenced by machine configuration, temperature, and valve geometry, while the cell resistance constant a is dependent on microbial characteristics such as growth rate, morphology, and physiology [43, 46].

The homogenizer has also been used to study the release of enzymes E (µmoles/min/g yeast) and protein R (mg protein/g yeast) from the *S. cerevisiae*, with the equation being as follows:

$$E=\frac{C_{E}F}{C_{y}}\;\mathrm{and}\;\;\;R=\frac{C_{R}F}{C_{y}}$$

where *C_E_* and *C_R_* are enzyme activity and protein concentration in disrupted yeast cell suspension, *C_y_* initial cell concentration, and *F* is the fraction of cells disrupted. Optimal release occurred at 30°C and a higher pressure of 460 kg/mL [48]. Release profiles were time‐ and pressure‐dependent and varied based on subcellular localization. Proteins on the cell membrane or wall were released faster than cytoplasmic components [48, 49].

Early studies show that the mechanism for the disruption of cells in homogenization is the combination of one or more effects, such as shear forces in liquid and valve faces, impact against the side walls, velocity changes, turbulence, cavitation forces, turbulence velocity gradient of pressure (velocity of pressure drop) [50].

**FIGURE 7:**
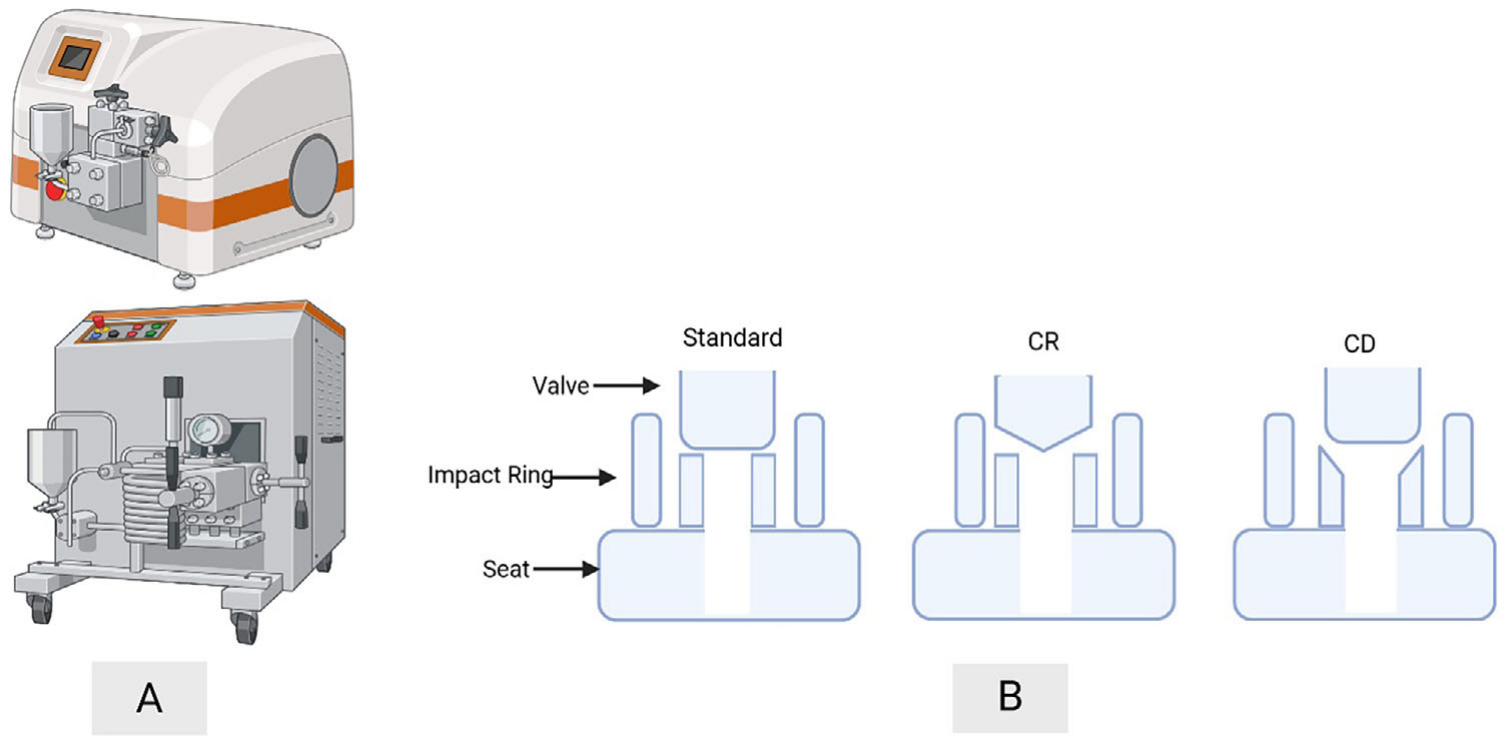
Homogenizer (A) laboratory scale and industrial scale and (B) different types of valve seats (CR, cell rupture with coned valve rod; CD, cell disruption) [52].

However, later work emphasized the role of impingement forces, especially near the valve rod, which is shown in Figure 7 and impact ring. Normal and elongational stresses were found to be less effective [44, 46, 51]. The stagnation pressure (*P_s_*) of the impinging jet, correlating with disruption, is described by:

$$P_{s}=\frac{1}{2}\;PV^{2}$$

where *P* is the cell suspension density and *V* is the jet velocity.

Later, a wall strength model was developed, taking into consideration that a single cell is disrupted when the disruptive forces exceed the effective wall strength and as well as the feed properties. The model can be used for multiple passes as follows:

$$D=1-\;\int_{0}^{\infty }{\left(1-f_{D}\left(S\right)\right)}^{N}f_{s}\left(S\right)dS$$

The degree of disruption *D* depends on the effective cell stability *S*, where $f_{D}\left(S\right)$ is stress distribution function of homogenizer and $f_{s}\left(S\right)\;$ is the strength distribution of the cells.

The $f_{D}\left(S\right)$ and $f_{s}\left(S\right)\;$ can be expressed as:

$$f_{D}\;\left(S\right)=\frac{{\left(mP^{n}\right)}^{d}}{S^{d}+{\left(mP^{n}\right)}^{d}}$$

$$f_{s}\;\left(S\right)=\frac{1}{\sigma \sqrt{2\pi }}\;\exp\neq \left(\frac{-{\left(S-S^{*}\right)}^{2}}{2\sigma^{2}}\right)$$

The parameters m, P, and n are not cell type or microorganism‐dependent but system‐specific. σ is the variance distribution of cell stability, S* is the mean effective strength of the culture, which is correlated with measurable properties of the feed cells, such as mean cell length, L, and fractional peptidoglycan cross‐linkage as X [46, 53].

The maximum stagnation pressure was also found to be impacting the group $kP^{a}$. And it was also related to the valve gap *h*, and impact distance *Y* as follows [51]:

$$P_{s}=\frac{1}{Y^{2}h^{2}}$$

Spiden [47] critically analyzed and compared all the disruption quantification methods for high‐pressure homogenizer and simplified the model of Hetherington as:

$$X_{t}=A^{N}$$

where *X_t_* is the proportion of disruption and *N* is a number of passes, *A* is the pressure‐dependent constant, which is a function of pressure. They also categorized HPH disruption mechanisms into four outcomes: (1) cells can remain as whole, (2) become damaged and release internal metabolites while remaining as whole, (3) break and release internal metabolites, and (4) complete fragmentation, which can be seen as depicted in Figure 9 [47].

**FIGURE 8:**
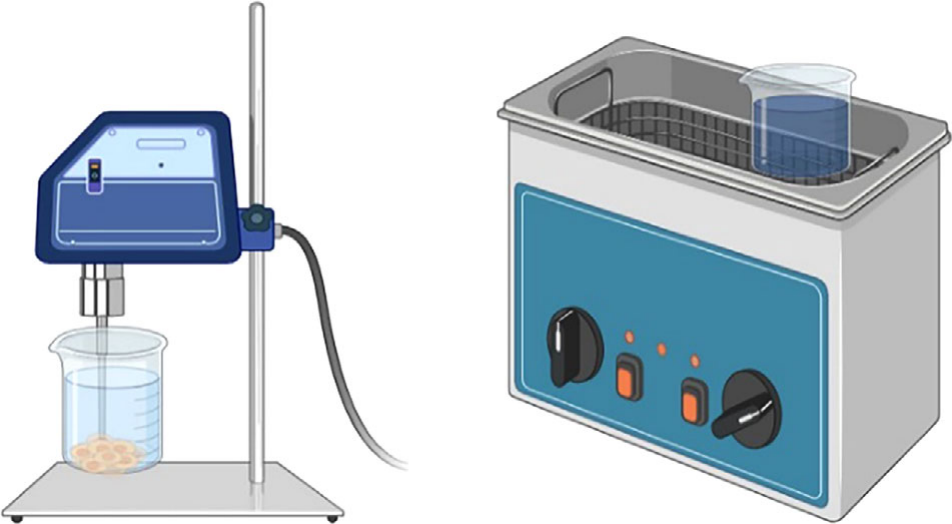
Ultrasonic equipment‐probe and bath.

The number of passes required to break the cells through the homogenizer is a critical factor for effective cell disruption. Typically, 4–5 passes are required to achieve complete disruption of cells, depending on the organism and applied. For yeasts, pressure above 80 to 150 MPa generally require 1–5 passes to reach satisfactory disruption level. In contrast, filamentous or pelleted cells may be disrupted in a single pass either at 50 or 10 MPa, although 2–3 passes are often required for maximal product release [45, 47, 54, 55, 56].

Oleaginous fungi such as *Yarrowia lipolytica* tend to require significantly more energy input, often needing 5–20 passes for efficient disruption [57, 58, 59].

For the highest protein yield from yeast, it was seen that it required 10 cycles at 120 MPa, whereas when it was combined with pH shifting, higher yield was achieved around 65% in 4 cycles above or around 100 MPa [60].

There is a required threshold pressure below which little to no disruption occurs. For yeasts, this threshold is in the range of 115 to 200 bar [54, 61]. For oleaginous and filamentous fungi, the pressure required for cell disruption is generally higher, ranging from 400 to 1500 bar, due to their more robust cell wall structures [57, 62, 63].

In general, it can be seen that high‐pressure homogenizer has been used across a range of fungal systems, from unicellular yeasts to filamentous and oleaginous species. Some comparative analyses for fungi and yeast are presented in Table 2. Disruption kinetics vary depending on morphology. In yeasts, maximum protein release *R*_max_ is strongly pressure‐dependent. In contrast, for filamentous fungi, *R*_max_ is only weakly influenced by pressure and may instead rely more on pass number, cell aggregation state, or wall rigidity.

**TABLE 2:** Comparison of high‐pressure homogenization parameters for fungal cell disruption.

Cell type	Fungal species	Target product	Biomass concentration (g/L)	Pressure (MPa or bar)	No. of passes	Pretreatment applied	Temperature control (Yes/No)	Disruption efficiency /Yield (%)	Ref.
Unicellular	*Saccharomyces cerevisiae*	Beta glucan	NM	800 bar	3	High temperature	Yes	NM	[54]
Unicellular	*Saccharomyces cerevisiae*	Protein	100 g/L	120 MPa	10	No	No	35%	[60]
Unicellular, oleaginous yeast	*Yarrowia lipolytica*	Lipid	150 g/L (Dry mass)	1500 bar	6	No	No	100%	[57]
Unicellular, oleaginous yeast	*Yarrowia lipolytica*	Oil	150 g/L	1500 bar	20	Applied it as pretreatment	Yes	100%	[59]
Filamentous	*Rhodosporidium toruloides* NCYC 921	Lipid/carotenoids	NM	600 bar	3	No	No	55.9%	[64]
Filamentous	NM	Protein	40 g/L	900 bar	2	No	No	77%	[63]

#### 3.1.3 Ultrasound‐Assisted Cell Disruption

Ultrasound has been widely used for the disruption at the laboratory scale. It typically employs high‐frequency sound waves ranging from 15 to 40 kHz. A piezoelectric transducer converts alternating current into mechanical waves, which are transmitted to the sample. During ultrasonication, the gas and vapor bubbles form in the liquid medium and within the cells. These bubbles grow and collapse, turning sonic waves into mechanical energy, a process known as cavitation, which results in cell wall breakage. Different types of ultrasonic equipment, such as a probe and a bath, are shown in Figure 8.

**FIGURE 9:**
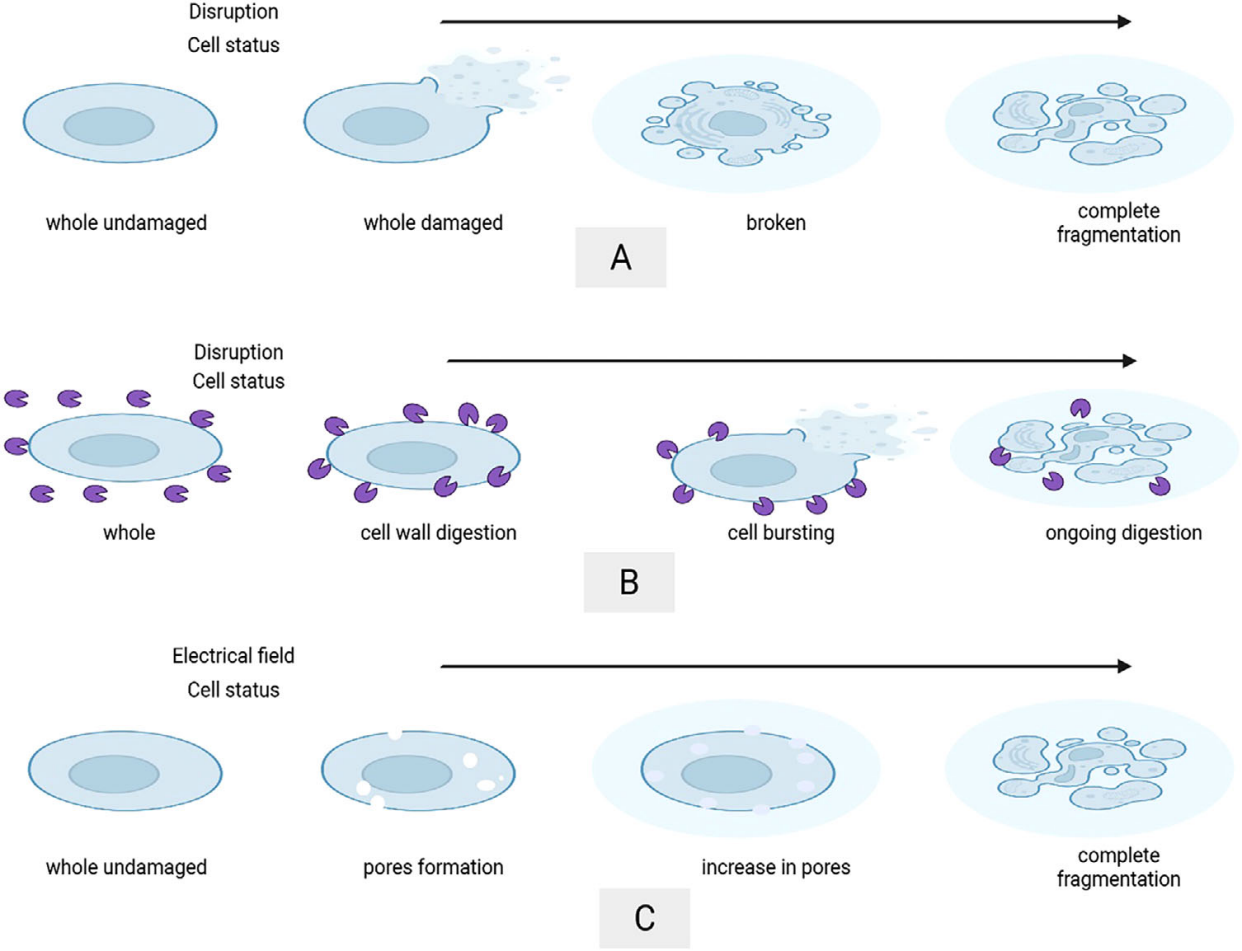
Mechanism of cell disruption; (A) In mechanical (high pressure homogenizer), (B) In non‐mechanical (enzymatic), and (C) In non‐mechanical (electrical).

The factors influencing the efficiency of cell disruption are amplitude or intensity, temperature, cell concentration, frequency, vessel capacity, and shape of the probe (horn or bath).

The protein release during ultrasonication follows first‐order kinetics:

$$\frac{R_{t}}{R_{\max}}=1-\exp\left(-k*t\right)$$

where *R*_max_ is the maximum protein release, *R_t_* is the protein release at time *t*, and *k* is the kinetic coefficient for protein release rate constant.

The *R_t_* can also be calculated based on the protein mass released during sonication as follows:

$$R_{t}=100*\frac{y_{t\;}-y_{b}}{y_{tot}-y_{b}}$$

where in the $y_{t\;}$ is the soluble protein mass concentration at sonication time *t*, $y_{b}$ is the background protein mass concentration and $y_{tot}\;$ is the total protein mass concentration [65]. The rate constant *k* is affected by the parameters such as acoustic power and duty cycle. The dependency on acoustic power follows:

$$k\;\propto {\left(W-\;W_{0}\right)}^{\alpha }\;\mathrm{or}\;k\;\propto W^{\alpha }$$

where *W* is the acoustic power, W_0_ is the cavitation threshold, and α is a constant. For yeast, α values of 0.9 and 0.52 have been reported [65, 66]. Higher acoustic power increases protein release. For example, 600 W yields nearly double the protein release compared to 120 W in yeast. Similar trends have been observed for polysaccharide release from *Cordyceps sinensis* and *S. cerevisiae* [65, 67, 68, 69, 70].

However, saturation occurs beyond certain power levels. For yeast, saturation is seen around 80 W, where further increase may cause protein denaturation or equilibrium between release and degradation [71].

Sonication time also influences protein and enzyme release, but can cause denaturation if extended. The duty cycle, which is the ratio of sonication time on to sonication time off, also impacts the rate constant:

$$k=D^{\beta }$$

where *D* is the duty cycle, and *β* is the constant, which is 1.0 for the yeast cells [65]. Duty cycle has a stronger effect on *k* than acoustic power. Combined, their relationship is:

$$k=k_{0}\;P^{\alpha }*\exp\neq \left(\beta \;.\;D\right)$$

where *k_0_* is a pre‐exponential constant, *P* is acoustic or ultrasonic power, and α and *β* are constants. For yeast, *α* = 0.3558, *β* = 0.01, and *k_0_* = 5.545 × 10^5^ [67]. An 80% duty cycle resulted in significantly higher protein release than 10% [65, 67].

The effect of cell concentration is inconsistent. Some studies (30–150 g/L) show little change in protein release, while others (3.3–333 g/L) show decreased release at higher concentrations [65, 69, 70, 71].

Temperature is another critical factor. Protein release increases from 5°C to 25°C–30°C, then decreases at higher temperatures due to denaturation [70]. For polysaccharides, higher temperatures up to 85°C improve yield [68, 69, 70, 71].

pH and salt concentration also affect biomolecule release. Protein and polysaccharide release improve from pH 5 to 7 but plateaus or decreases at pH 9. High salt concentrations reduce protein and polysaccharide release, likely due to plasmolysis and decreased cell size [65, 69]. High salt concentrations reduce protein and polysaccharide release, likely due to plasmolysis and decreased cell size [69, 70].

Ultrasound device type impacts efficiency. Horn‐type probes induce more intense cavitation than bath‐type systems, which are milder. Baths require a longer time and higher frequency to achieve similar yields [67, 71].

Novel geometries such as Barbell Horn Ultrasonic Technology and compact tubular transducers have been tested on lab and pilot scales for disrupting yeast [72, 73]. A fully automated sonication robot has also been developed for filamentous fungi, demonstrating that higher power and temperature significantly enhance disruption [74].

Even within yeast, different optimization strategies are needed to maximize the release of enzymes, proteins, and polysaccharides, due to their intracellular localization and the physical nature of cavitation‐induced shear stress [49, 70, 75, 76, 77]. Ultrasonication has also been applied to release lipids from *Trichosporon* spp., enzymes and carotenoids from *Candida tropicalis, Sporidiobolus pararoseus*, *Rhodotorula mucilaginosa* and *Xanthophyllomyces dendrorhous*, and proteins from *Leucosporidium muscorum* and *Aspergillus terreus* (CCT 7693) [78, 79, 80, 81, 82]. Recently, ultrasound has been used to enhance the autolysis of yeast in fermentation and to recover ingredients for the food industry [83, 84].

In general, ultrasonic cell disruption is a well‐established technique for laboratory‐scale fungal biomass processing, ranging from 0.5 mL to a maximum of 1000 mL. It is especially effective for unicellular fungi like yeast, where kinetics have been extensively studied and often follow first‐order models. However, further research is needed to understand its behavior in filamentous fungi, which may not follow the same kinetic patterns due to structural differences. Despite its effectiveness at small scales, ultrasound has not yet been widely adopted for large‐scale industrial applications. Some of the comparative analyses for fungal cell disruption using the ultrasonication methods are presented in Table 3.

**TABLE 3:** Comparison of ultrasonic parameters for fungal cell disruption.

Cell type	Fungal species	Target product	Biomass concentration (g/L)	Amplitude (%), Intensity (W/cm^2^) or frequency (Hz/kHz)	Time (min)	Temperature control (Yes/No)	Disruption efficiency (%) yield	Ref.
Unicellular	*Saccharomyces cerevisiae*	Enzyme	400 g/L	62%	30 min	No	NM	[75]
Unicellular	*Saccharomyces cerevisiae*	Protein and polysaccharides	200 g/L	39 W/cm^2^	30 min	Yes	84%–92%	[70]
Filamentous	*Aspergillus terrus*	Enzyme	25 g/L	40%	7 min	No	NM	[81]
Unicellular yeast	*Xanthophyllomyces dendrorhous*	Carotenoids	12.5 g/L (dry mass)	40 kHz	56 min	Yes	89%	[80]
Oleaginous yeast	*Trichorosporon* spp.	Lipids	5 g/L (dry mass)	50 Hz	20 min	Yes	95%–97%	[78]
Unicellular yeast	*Rhodotorula mucilaginosa*	Carotenoids	83 g/L (dry mass)	40 kHz	10 min	Yes	72%	[82]

### 3.2 Non‐Mechanical Cell Disruption

#### 3.2.1 Electrical Method

Electrical cell disruption includes techniques such as high‐voltage electric discharges and pulsed electric fields (PEF). In high‐voltage discharge methods, electric fields up to 40 kV and 10 kA are applied through electrodes, generating cavitation and turbulence that lead to cell rupture and intracellular product release. The efficiency of this process depends on several parameters: electric field strength, electrode gap, treatment time, temperature, solvent composition, and solvent‐to‐cell ratio [85].

PEF is a non‐thermal technique where disruption occurs due to electrical breakdown and electroporation of the cell membrane. The process can be divided into the following stages: (1) increase in transmembrane potential due to the applied electric field, (2) formation of small pores, (3) expansion of pores, and (4) release of intracellular contents [86, 87] (Figure 9).

In PEF, the electric field strength is a critical factor, influencing both extraction efficiency and the physical properties of the released compounds. Uniform field distribution is essential and is often achieved using bipolar, oscillatory, or square pulses—the latter being preferred due to their high energy and effectiveness [88].

Optimizing electric field intensity is key: lower fields require longer treatment times and can reduce product yield, while excessively high fields may damage biomolecules. For S. cerevisiae, permeability increased between 10 and 20 kV/cm, enhancing the release of ions, proteins, and nucleic acids [56, 89, 90]. However, beyond 20 kV/cm, yields often declined, likely due to cellular degradation.

In mushrooms such as *Agaricus bisporus*, *Lentinula edodes*, and *Pleurotus ostreatus*, lower field strengths (e.g., 2.5 kV/cm) were sufficient for maximizing the release of bioactive compounds [91, 92].

Continuous PEF treatment of *A. bisporus* achieved the highest polysaccharide and polyphenol yields at 38.4 kV/cm, while protein yields declined. For *Pleurotus pulmonarius*, no further increase in β‐glucan yield was observed beyond 7.5 kV/cm [89, 93, 94, 95].

Temperature also affects PEF efficiency. Optimal performance is usually found between 40°C–50°C. Lower temperatures can slow down the extraction process, while higher temperatures may denature sensitive molecules. For example, polysaccharide yield from mushrooms increased from 4°C to 73°C, whereas protein yield from yeast peaked at 45°C before declining [56, 93, 95].

Treatment time, expressed as pulse number or width, influences pore size and the extent of disruption. Initially, increased treatment time enhances yield, but excessive exposure may cause structural damage and reduce product recovery. In yeast, pulse numbers between 2 and 6 maximized protein release, with longer treatments showing diminished returns [89, 96]. Similar effects were observed in brewer's yeast for pulse durations ranging from 20 to 150 µs [90], and for mushroom carbohydrates, prolonged stirring was required for up to 6 h was needed [91, 92].

Solvent choice also impacts PEF performance. Solvents affect conductivity, polarity, and membrane interaction. For yeast, citrate‐phosphate McIlvaine buffer (2 mS/cm) [56, 90], while some used Milli‐Q or deionized water for mushroom and yeast with a conductivity of 0.7 to 1 mS/cm [89, 91, 92, 93, 96].

Cell concentration and solid‐to‐liquid ratio also play roles but show conflicting effects. Ratios from 1:20 to 1:60 have been tested, with optimal trehalose yields at 1:30 or 1:50 depending on the target molecule [89, 96]. For yeast protein extraction, concentrations from 12.5 to 85 g DCW/L showed little effect [95].

PEF has been compared with other methods. For lipid extraction from *Yarrowia lipolytica*, PEF achieved 92.86% efficiency, while high‐voltage electrical discharges (HVED) achieved even higher yields in some studies [58]. HVED also showed superior extraction performance in *S. cerevisiae* (bayanus) [97]. PEF is a low‐energy input, whereas HVED uses high‐energy input for cell disruption, as HVED treatment damages cell membranes better than PEF [97].

Additionally, PEF has been used to accelerate yeast autolysis. Higher electric field strength and longer treatments increased cell disintegration but sometimes reduced the release efficiency of intracellular contents [98]. Currently, electrical methods like PEF are primarily used for unicellular and oleaginous yeasts. Their application to filamentous fungi remains limited, and further studies are needed to understand their kinetics and efficacy in these systems.

#### 3.2.2 Physical Methods

Physical disruption techniques include freeze‐thawing, osmotic shock, thermolysis, and decompression. In freeze‐thawing, cells are repeatedly frozen at −20°C to −40°C and thawed, leading to the formation of the ice crystals which rupture the membrane structure. Osmotic shock involves sudden changes in salt or sugar concentration in the outside environment. It can also be a pH shock where there is a change to acidic or alkaline pH [18, 24].

Typically, cells are first exposed to a high‐osmolarity solution (≥1 M) followed by transfer to a low‐osmolarity environment, causing water influx and lysis. Thermolysis involves exposure to heat shock, while decompression uses pressurized gas release to rupture cells. These methods are predominantly applied at the laboratory scale. However, autolysis, a related self‐degradation process, is used industrially for yeast biomass processing, such as in winemaking.

Osmotic shock and freeze‐thawing have been tested for the disruption of cell walls of *Cyberlindnera jadinii* and *Rhodotorula glutinis*  yeast cells to enhance lipid extraction. However, neither method improved yields compared to controls [99]. Similarly, osmotic shock was ineffective for protein release from *Penicillium citrinum, A. fumigatus*, and *Rhodotorula gracilis* [100]. Freeze‐thawing as a pretreatment for lipid extraction from *Yarrowia lipolytica* resulted in lower yields than conventional methods, indicating limited efficacy [101].

The osmotic shock is mostly effective for the cells with weak walls, like blood cells, or some bacteria, but less effective for fungi. Both osmotic shock and freeze‐thawing pose scalability issues and risk damage to intracellular biomolecules, particularly due to ice formation. Their reproducibility is also limited, restricting their use mostly to small‐scale applications.

Although these techniques have long been used in cell disruption, research has primarily focused on yeasts such as baker's or oleaginous strains. Further studies are needed to evaluate the response of filamentous fungi, particularly with their hyphal morphology, to these physical methods. Some of the comparative analyses for fungi physical cell disruption methods are mentioned in Table 4.

**TABLE 4:** Comparison of physical parameters for fungal cell disruption.

Cell type	Fungal species	Target product	Method used	Biomass concentration (g/L)	Time (h)	Temperature control (Yes/No)	Disruption efficiency/Yield (%)	Ref.
Unicellular	*Cyberlindnera jadinii* ATCC 9950	Lipids	Osmotic shock	20 g/L (dry mass)	48 h	Yes	11.93	[99]
Unicellular	*Cyberlindnera jadinii* ATCC 9950	Lipids	Freezing/Defrosting	20 g/L (dry mass)	72 h	Yes	13.74	[99]
Unicellular, oleaginous yeast	*Rhodotorula glutinis* LOCKR13	Lipids	Osmotic shock	20 g/L (dry mass)	48 h	Yes	14.10	[99]
Unicellular, oleaginous yeast	*Rhodotorula glutinis* LOCKR13	Lipids	Freezing/Defrosting	20 g/L (dry mass)	72 h	Yes	15.84	[99]
Filamentous	*Aspergillus terrus*	Enzyme	Osmotic shock	12.5 g/L	0.3 h	Yes	Failed	[81]
Mushroom	*Pycnoporus cinnabarinus*	Enzyme	Freezing/Defrosting	50 g/L	3 h	Yes	NM	[27]
Mushroom	*Ganoderma applanatum*	Enzyme	Freezing/Defrosting	50 g/L	3 h	Yes	NM	[27]

#### 3.2.3 Enzymatic Methods

Enzymatic cell disruption uses specific enzymes to hydrolyze microbial cell walls, offering a milder, greener, and lower‐energy alternative to mechanical or chemical methods. It involves enzymatic digestion of cell wall components, enabling the release of intracellular products. Key parameters influencing disruption efficiency include temperature, pH, incubation time, enzyme concentration, and particle size. These factors must be optimized depending on the organism and cell wall composition. Enzyme cocktails are often employed, as no single enzyme can completely degrade fungal cell walls. Commonly used enzymes include cellulases, hemicellulases, chitinases, and proteases [4, 102, 103].

The kinetics of released protein and carbohydrate for the yeast were studied dating back to 1987. The equation was developed in accordance with Michaelis–Menten equation, which is as follows

$$\frac{dP}{dt}=-f_{py}\left(\frac{dY}{dt}\right)-\frac{k_{p}E\;P}{P+S+K_{mp}\left(1+\frac{Y}{K_{i}}\right)}$$

$$\frac{dC}{dt}=-f_{cy}\left(\frac{dY}{dt}\right)$$

Here, P, S, and C are the protein, peptides, and carbohydrates (mg/L); *Y* is yeast concentration, and E is the enzyme used (% v/v); *k*_p_ is the rate constant for the lysis, *K*_mp_ is the Michaelis constant for protein lysis; *f*_py_ and *f*_cy_ are the fractions of proteins and carbohydrates in yeast, and *k_i_* is the inhibition constant. The lytic systems of Cytophaga and Oerskovia were evaluated, with Cytophaga demonstrating higher lysis efficiency across yeast concentrations of 0.7–70 g/L and enzyme doses of 4%–40% v/v [104, 105, 106].

The mechanism for the enzymatic cell lysis occurs in three stages: (1) Enzymatic degradation of the two‐layered cell wall, (2) Cell bursting under osmotic pressure differences, and (3) Digestion of released intracellular contents by residual enzymes (Figure 9).

Monte Carlo simulations have been used to model the second step [107]. Combining enzymatic hydrolysis (e.g., zymolyase) with mechanical disruption enhances efficiency. For instance, combining zymolyase with high‐pressure homogenization (95 MPa, 4 passes) achieved 100% yeast disruption, compared to 32% with homogenization alone. This synergy was also effective for *Candida utilis* [108, 109].

Several operational parameters influence enzymatic lysis. The pH of the reaction medium plays a critical role in maintaining enzyme activity. Most enzymes used in cell disruption, such as alcalase, viscozyme, Flavourzyme, lyticase, and papain, are active in a neutral pH range of 5 to 8. However, others, such as β‐glucanase, perform optimally under mildly acidic conditions, around pH 4.5 [110, 111].

Temperature is equally important, as enzyme activity can be diminished or lost entirely if the conditions deviate from their optimal range. Most enzymes are effective between 20°C and 50°C, though some, such as alcalase, papain, and lyticase, show maximum activity around 60°C [110, 111, 112, 113, 114].

The enzyme concentration must also be carefully optimized. Typically, concentrations range from 0.01% to 1% (v/v or w/w), depending on the enzyme and target organism. For example, papain concentrations of 2.5% v/v yielded higher amounts of solids, proteins, and carbohydrates from yeast extract than 0.5%, while lyticase was effective at just 0.025% v/v [110]. Alcalase was used at 0.2% v/v for yeast disruption [112], and alkaline protease was applied at 3% v/v for Schizochytrium species [113]. In addition to enzyme concentration, enzyme activity should also be considered, as activity‐based units (U/mL) provide a more accurate indication of the catalytic performance of the enzyme preparation and therefore offer a standardized and more meaningful basis for evaluating its effectiveness in fungal cell disruption.

Incubation time is another crucial factor, as prolonged exposure may result in the degradation of target molecules. The optimal duration depends on the organism and product. For *Schizochytrium*, 9 to 10 h was sufficient for high yields, while for yeast, disruption times of up to 72 h have been reported [110, 112, 113].

Enzymatic disruption has also been applied to extract bioactive compounds from fungal biomass. In mushrooms such as *Hericium erinaceus* and *Ganoderma lucidum*, enzymes like β‐glucanase, Flavourzyme, viscozyme, chitinase, and cocktails containing cellulase, pectinase, and trypsin or papain have been used to release amino acids, polysaccharides, and triterpenoids [115, 116, 117, 118, 119, 120]. In yeast extract production from brewer's yeast, combinations of endoprotease, exoprotease, 5′‐phosphodiesterase, and AMP‐deaminase have been used to improve solubilization [121].

In general, enzyme‐based disruption is a promising approach when working with sensitive biomolecules that could be damaged by harsher methods. Although it is a scalable and gentle technique, its broader industrial application is currently limited by the high cost of enzymes. While the kinetics of enzymatic lysis have been extensively studied in yeast, further investigations into filamentous fungi are needed. Some studies on fungal enzymatic cell disruption are listed in Table 5. The complex hyphal structures of different fungi present both challenges and opportunities for expanding the use of enzymatic disruption in fungal biotechnology. The type and concentration of the enzyme used in the studies are compared in the table below. However, most referenced studies report only enzyme concentration, and activity values were not available. Consequently, enzyme activity could not be consistently included in Table 5.

**TABLE 5:** Comparison of enzymatic parameters for fungal cell disruption.

Cell type	Fungal species	Target product	Biomass concentration (g/L)	Enzymes	Enzyme conc. (v/v), (w/w) or %	Time (h)	Temperature control (Yes/No)	Yield (%)	Ref.
Unicellular	*Saccharomyces cerevisiae*	Yeast extract	150 g/L	Alcalase	0.2 (w/w)	48 h	Yes	27.9 %	[112]
Unicellular	*S. cerevisiae*	Canthaxanthin	NM	Alcalase,Viscozyme, Promozyme	2–3 (v/v)	4 h	Yes	39.5 %	[122]
Mushroom	*Lentinus edodes*	Umami taste amino acids	10 g/L	*β*‐Glucanase‐Flavourzyme	5 (v/v)	1 h	Yes	—	[116]
Mushroom	*Ganoderma lucidum*	Polysaccharide	50 g/L (Dry mass)	Viscozyme and chitinase	3 %	0.5 h	Yes	32 %	[123]
Unicellular	*Saccharomyces cerevisiae*	Beta glucan	NM	Alcalase, Flavourzyme, Viscozyme, neutral Protease ST, or papain	0.1 %	0.5 h	Yes	—	[124]

#### 3.2.4 Chemical Methods

Chemical cell disruption involves the use of acids, alkalis, detergents, or organic solvents to compromise the integrity of microbial cell walls or membranes. This approach is generally considered milder than mechanical techniques, as it selectively interacts with cell wall components or membrane lipids to release intracellular biomolecules into the surrounding medium. Commonly used chemical agents include hydrochloric acid (HCl) as an acid, sodium hydroxide (NaOH) as an alkali, and various detergents such as Triton X‐100, CTAB, Tween‐80, urea, EDTA, and guanidinium salts. Organic solvents like ethanol, toluene, acetone, butanol, and isopentanol can also be employed to disrupt lipid‐rich membranes by increasing their permeability and facilitating product release.

Chemical methods have been successfully applied for the extraction of carotenoids and lipids from fungal cells. For example, organic solvents such as dimethyl sulfoxide (DMSO), petroleum ether, acetone, chloroform, hexane, and ethyl acetate were evaluated for carotenoid extraction from red yeasts *Rhodotorula glutinis* and *Sporidiobolus salmonicolor*. DMSO yielded the highest carotenoid content for both species, while petroleum ether and acetone were also effective for *R. glutinis*. Notably, the combination of DMSO with liquid nitrogen resulted in the highest overall yield [125, 126].

For lipid extraction, chemical disruption methods have been applied to various oleaginous yeasts and fungi, including *Cryptococcus curvatus*, *Mortierella isabellina*, and *Yarrowia lipolytica*. In these cases, treatments with hydrochloric acid or detergents such as Triton X‐100 resulted in higher lipid yields compared to physical disruption methods [127, 128]. Similarly, acid hydrolysis using HCl was effective for *Cyberlindnera jadinii* ATCC 9950 and *Rhodotorula glutinis* LOCKR13. For filamentous fungi such as *Penicillium citrinum, A. fumigatus, and Rhodotorula gracilis*, detergents like CTAB and SDS proved ineffective, while Triton X‐100 (at 5%) and Tween showed limited success [99, 100]. In another study, a combination of lithium acetate and sodium hydroxide was found to enhance protein release from yeast cells, demonstrating that chemical disruption efficacy can be improved through reagent synergy [129].

Despite their potential, chemical methods are not widely used at an industrial scale due to several drawbacks. These include the risk of altering or denaturing sensitive biomolecules, difficulties in removing chemical residues, and challenges in process scalability. Nonetheless, they remain valuable at the laboratory scale for studying cell wall composition, permeability, and fundamental lysis mechanisms. Most studies to date have focused on yeast, while applications to filamentous fungi remain limited, largely due to the structural complexity and variability of fungal cell walls, which makes a one‐size‐fits‐all chemical approach impractical. However, some available studies on chemical cell disruption of fungi are compared in Table 6.

**TABLE 6:** Comparison of chemical parameters for fungal cell disruption.

Cell type	Fungal species	Target product	Chemicals used	Biomass concentration (g/L)	Conc. (v/v) or molar	Time (h)	Temperature control (Yes/No)	Yield (%)	Ref.
Unicellular	*Cyberlindnera jadinii* ATCC 9950	Lipids	Acid hydrolysis (HCl)	20 g/L (dry mass)	1 molar	2 h	Yes	20.37%	[99]
Unicellular, oleaginous yeast	*Rhodotorula glutinis* LOCKR13	Lipids	Acid hydrolysis (HCl)	20 g/L (dry mass)	1 molar	2 h	Yes	21.20 %	[99]
Unicellular, oleaginous yeast	*Yarrowia lipolytica*	Lipids	Detergent (Triton X‐100)	NM	5%	1 h	NM	NM	[128]
Unicellular, oleaginous yeast	*Cryptococcus curvatus* (ATCC 20509)	Lipids	Acid hydrolysis (HCl)	50 g/L (dry mass)	4 molar	2 h	Yes	47.30%	[127]
Unicellular	*Mortierella isabellina* (NRRL 1757)	Lipids	Acid hydrolysis (HCl)	50 g/L (dry mass)	4 molar	2 h	Yes	54.8%	[127]
Unicellular, oleaginous yeast	*Rhodotorula glutinis* KCTC7989	Carotenoids	Solvent hydrolysis (DMSO, petroleum ether, acetone, NaCl)	4.3 g/L (dry mass)	34:16:18:31 (%)	NM	Yes	0.026%	[125]
Unicellular	*Saccharomyces cerevisiae*	Protein	Chemical hydrolysis (lithium acetate and NaOH)	NM	2:0.5 molar	0.16 h	Yes	NM	[129]

#### 3.2.5 Newer (Other) Methods

There has been a continuous effort to identify cell disruption methods that are not only more efficient but also greener and more sustainable. Various approaches have been studied and compared, particularly in yeast, to enhance the release of intracellular products. For instance, Duarte [130] evaluated different techniques for lipid extraction and found that shear abrasion was the most effective method for disrupting yeast cells. Michelon compared mechanical, chemical, and enzymatic methods for carotenoid extraction from *Phaffia rhodozyma*, concluding that maceration combined with diatomaceous earth and enzymatic lysis produced the highest carotenoid yields [111].

Innovative and non‐contact techniques have also been explored. Wenger studied adaptive focused acoustics as a non‐invasive method for disrupting small quantities of yeast cells to purify virus‐like particles [131]. Thakkar introduced a novel method known as flash hydrolysis for yeast disruption and protein recovery and also characterized the associated kinetic parameters [132].

Comparative studies of industrial‐scale methods have been conducted as well. Jacob investigated the effectiveness of a cell mill, sonotrode, and autolysis for yeast disruption in relation to amino acid recovery [133]. Autolysis emerged as the most effective, but the other two methods were deemed suitable alternatives when preservation of valuable nutritional components was required. Hydrodynamic cavitation, an emerging technique similar to ultrasound, has shown promising potential for large‐scale applications. Mevada reported that combining hydrodynamic cavitation with pretreatments such as acidic stress, hypoosmotic conditions, and reducing agents significantly improved selectivity (by a factor of 4.79) and energy efficiency (by 63.19 times) compared to high‐pressure homogenization [134].

Other researchers have explored hybrid techniques that combine mechanical and electrical disruption. Shynkaryk studied the electrically assisted high‐pressure homogenization of wine yeast cells, incorporating pulsed electric fields (PEF) or high‐voltage electric discharge (HVED) to enhance disruption efficiency [97].

### 3.3 Scalability and Comparison of Different Disruption Methods

For any cell disruption method to be industrially relevant, scalability is a crucial factor. While various techniques can be tested and optimized at laboratory scale, their transition to industrial application presents significant challenges. Methods must not only be effective but also economically viable, energy‐efficient, and compatible with downstream processing.

Mostly mechanical disruption methods such as high‐pressure homogenizers, French press, or bead/ball milling are used at scale due to their established efficiency, though they are typically associated with medium to high energy consumption. In contrast, non‐mechanical approaches such as pulsed electric fields (PEF), chemical treatments, and enzymatic methods are gaining interest for their potential selectivity and gentleness. However, their industrial use remains limited. Enzyme‐based disruption, for example, offers high specificity and product integrity but is often cost‐prohibitive, restricting its large‐scale application to niche areas like autolysis in yeast extract production or fermentation.

Several studies have demonstrated that combining different disruption methods can yield better results than using a single technique alone. For instance, enzymatic pretreatment combined with bead milling significantly enhanced cell disruption efficiency, achieving nearly 100% disruption compared to ∼45% with bead milling alone [135]. Similarly, for the release of β‐glucans from *Saccharomyces cerevisiae*, a comparison of various methods, including hot water treatment (autoclaving), thermally induced autolysis, bead milling, and sonication, showed that bead milling with 1 mm beads provided the highest β‐glucan yield [41].

For the extraction of mannans and glucans from yeast, the combination of bead milling and enzymatic treatment led to improved recovery compared to either method alone [136]. Comparable findings have been reported for filamentous fungi. In a study comparing various disruption strategies such as percussion grinding, Ultraturrax homogenization, and chemical treatment for *Ganoderma applanatum*, *Pycnoporus cinnabarinus*, and *Rhodotorula glutinis*, the most effective method varied by species. For *P. cinnabarinus*, percussion grinding yielded the highest efficiency, while *G. applanatum* responded best to a combination of percussion grinding with Ultraturrax pretreatment. In contrast, *R. glutinis* showed maximum carotenoid release with bead milling alone [27, 28].

These findings underscore the importance of tailoring disruption strategies to the specific organism and target product. Fungal species vary significantly in cell wall composition and structure, which influences their susceptibility to mechanical, chemical, or enzymatic lysis. Therefore, a universal approach is unlikely to be effective. Instead, application‐specific optimization, possibly involving synergistic combinations of methods, is necessary to achieve efficient and scalable cell disruption in fungal biotechnology. In Table 7, comparative analysis of these methods is made, where the key parameters, advantages, and disadvantages, as well as the energy and scalability of these methods, are mentioned.

**TABLE 7:** Comparative analysis of fungi cell disruption methods.

Method	Advantages	Disadvantages	Key parameters	Operation cost	Volume of operation	Industrial use and scalability	Effective organism or efficiency	Energy used	Challenges
Bead mill	Highly effective for breaking tough mycelial structures	High energy consumption, bead contamination	Bead size, agitation, time	Moderate	High	Yes	Effective but needs cooling for heat‐labile products	Moderate	Bead wear, contamination
High‐pressure homogeniser	Scalable, efficient	Requires multiple cycles for thick‐walled fungi	Pressure, flow rate	Moderate	High	Yes	Highly efficient but energy‐consuming	Moderate	Heat generation
Ultrasound	Effective in small‐scale applications	Heat generation, low scalability	Intensity	Low to moderate	Low to moderate	Limited	Effective for small‐scale	Low to Moderate	Heat buildup, aeration
Electrical	Non‐thermal, selective permeability preserves intracellular components	High voltage requirement, less effective on rigid cell walls	Voltage, pulse duration, field strength	Moderate to high	Low to moderate	Limited industrial scale, but growing in biotech	Effective for bacteria, yeasts, and less so for filamentous fungi	High	High energy demand, safety concerns, and low efficiency for tough walls
Physical	Simple, gentle, chemical‐free, mild conditions	Time‐consuming, not efficient for tough cell walls	Number of cycles, Osmolarity gradient, exposure time	Low	Low to Moderate	Suitable only at lab scale	Effective for mammalian cells, some yeasts	Low to Moderate	Not effective on fungi
Chemical	Breaks tough walls effectively	Harsh chemicals may degrade sensitive compounds	pH, temperature, type	Low	Low to moderate	Limited	low energy, but chemical wastage and disposal an issues	Variable	Environmental concerns
Enzymatic	Gentle, preserves intracellular products	Expensive, slow	Enzyme conc, pH, temperature	High	Low to moderate	Yes	Highly efficient but slow	Low	High enzyme cost

## 4 Conclusion

Obtaining intracellular biomolecules from fungi remains challenging due to the complexity and rigidity of their cell walls. Nevertheless, fungal biotechnology continues to advance, driven by its ability to produce diverse high‐value compounds and to grow efficiently on low‐cost and waste‐derived substrates. Efficient cell disruption, therefore, remains a pivotal step that directly influences product yield, purity, and overall process economics.

This review emphasizes that a comprehensive understanding of fungal cell wall architecture, morphology, and target product sensitivity is crucial for selecting an effective disruption strategy. Mechanical methods such as bead milling and high‐pressure homogenization are the most widely applied and offer high disruption efficiencies across yeast, filamentous, and oleaginous fungi. However, their high energy demand, heat generation, and potential product degradation limit their applicability for sensitive metabolites. Ultrasonication provides effective disruption at small scales but faces major scalability constraints. Among non‐mechanical approaches, enzymatic lysis is the most selective and gentle option, particularly for sensitive proteins and polysaccharides; however, cost and lengthy processing times remain significant drawbacks. Electrical, chemical, and physical methods show promise in specific contexts but currently lack broad applicability to filamentous systems.

In general, the comparison across techniques demonstrates that no single method is universally effective for all fungal species, mainly due to the pronounced structural diversity of fungal cell walls. This underscores the need for organism‐specific and product‐specific process optimization, as well as continued development of hybrid or integrated disruption approaches that balance efficiency, selectivity, and sustainability. Future work should focus on improving kinetic understanding, developing scalable low‐energy disruption technologies, and expanding research to underexplored fungal species. Such advancements will be essential for enabling robust, efficient, and sustainable fungal bioprocessing within a circular bioeconomy framework.

## Policy on Using ChatGPT and Similar AI Tools

The authors used ChatGPT (OpenAI) to improve the language of the manuscript.

## Funding

This study was supported by the German Academic Exchange Service (DAAD) grant.

## Conflicts of Interest

The authors declare no conflict of interest.

## Data Availability

This review summarizes previously published studies. No new data were generated, and all data referenced are available in the cited publications.
